# Clinical and microbiological effects of dialyzers reuse in hemodialysis patients

**DOI:** 10.1590/2175-8239-JBN-2018-0151

**Published:** 2019-01-24

**Authors:** Isabella Carvalho Ribeiro, Noemí Angelica Vieira Roza, Diego Andreazzi Duarte, Dioze Guadagnini, Rosilene Motta Elias, Rodrigo Bueno de Oliveira

**Affiliations:** 1 Universidade Estadual de Campinas Serviço de Nefrologia do Hospital de Clínicas CampinasSP Brasil Universidade Estadual de Campinas, Serviço de Nefrologia do Hospital de Clínicas, Campinas, SP, Brasil.; 2 Universidade Estadual de Campinas Faculdade de Ciências Médicas Departamento de Medicina Interna CampinasSP Brasil Universidade Estadual de Campinas, Faculdade de Ciências Médicas, Departamento de Medicina Interna, Campinas, SP, Brasil.; 3 Universidade de São Paulo Hospital das Clínicas São PauloSP Brasil Universidade de São Paulo, Hospital das Clínicas, São Paulo, SP, Brasil.; 4 Universidade Nove de Julho São PauloSP Brasil Universidade Nove de Julho, São Paulo, SP, Brasil.

**Keywords:** Renal Dialysis, Endotoxins

## Abstract

**Introduction:**

Chronic kidney disease (CKD) has a high prevalence and is a worldwide public health problem. Reuse of dialyzers is a cost reduction strategy used in many countries. There is controversy over its effects on clinical parameters and microbiological safety.

**Methods:**

In this clinical crossover study, 10 patients performed consecutive hemodialysis (HD) sessions divided in two phases: "single use" sessions (N = 10 HD sessions) followed by "dialyzer reuse" sessions (N = 30 HD sessions). Clinical, laboratory, and microbiological parameters were collected in the following time points: "single use", 1^st^, 6^th^, and 12^th^ sessions with reuse of dialyzers, including bacterial cultures, endotoxins quantification in serum and dialyzer blood chamber, and detection of hemoglobin and protein residues in dialyzers.

**Results:**

Mean age of the sample was 37 ± 16 years, 6 (60%) were men, and 5 (50%) were white. CKD and HD vintage were 169 ± 108 and 47 (23-111) months, respectively. Serum C-reactive protein (CRP) [4.9 (2.1) mg/mL], ferritin (454 ± 223 ng/mL), and endotoxin levels [0.76 (0.61-0.91) EU/mL] were high at baseline. Comparison of pre- and post-HD variations of serum levels of CRP and endotoxins in the "single use" versus "reuse" phases did not result in differences (*p* = 0.8 and 0.4, respectively). Samples of liquid in the dialyzer inner chamber were negative for the growth of bacteria or endotoxins. There was no significant clinical manifestation within and between the phases.

**Conclusion:**

Dialyzers reuse was safe from a clinical, microbiological, and inflammatory point of view. The dialyzer performance remained adequate until the 12^th^ reuse.

## Introduction

Chronic kidney disease (CKD) has high prevalence and is currently a worldwide public health problem. In Brazil, it is estimated that there are 122,825 patients on hemodialysis (HD), with approximately 39,000 new cases per year, whose treatment is mostly funded by the government.^1^ In the next decades, the increase in cases of diabetes mellitus and hypertension, added to the aging of the population will contribute to a higher prevalence of CKD, resulting in economic pressure on health systems around the world.^2^

Dialyzers reuse is a cost reduction strategy in many countries.^3^^-^6 However, the scientific evidence regarding the effects of reuse on clinical outcomes such as hospitalization or mortality, and repercussions on the environment are inconsistent.^4^^,^^7^^-^11

A systematic review involving 14 selected studies with a total of 956.807 patients evaluated the effects of dialyzer reuse or single dialyzer use on mortality. No consistent differences were identified, apart from important methodological limitations in the studies analyzed.^7^ In contrast, Lowrie et al. and Lacson et al. observed a trend of better survival in some groups of patients not exposed to dialyzer reuse.^4^^,^^11^

Some authors argue that the reuse of modern dialyzers does not interfere with the efficiency of dialysis treatment in terms of solutes clearance such as urea, phosphate and β2-microglobulin.^12^ Aspects involving a higher risk of biological contamination in patients exposed to dialyzers reuse are evidenced in other studies.^10^^,^^13^

Recently in Brazil, changes in the regulation regarding dialyzer reuse have been updated towards a greater restriction, associated with the requirement of local validation protocols for the reuse process.^14^ However, it is believed that the current governmental funding does not allow for partial or universal adoption of the single use of dialyzers. Thus, we estimated that most dialysis units in Brazil still reuse dialyzers.

Based on the inconsistency of results involving dialyzer reuse and the limitations of scientific information especially coming from the Brazilian scenario, we propose in this pilot clinical crossover study to evaluate the effects of single-use and reuse of dialyzers on clinical, microbiological, inflammatory, and dialysis efficiency parameters in prevalent hemodialysis patients.

## Methods

### Study design

**Figure 1:**
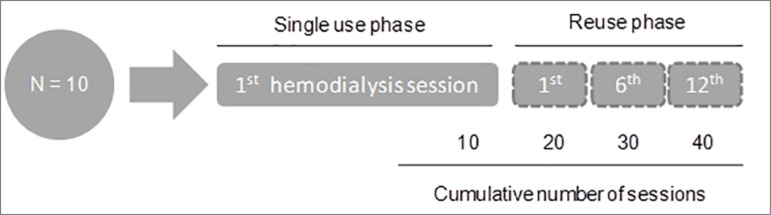
Study design.

This was a clinical crossover study conducted during September 2016 to February 2017, involving 10 patients undergoing HD treatment in the Nephrology Service at the Hospital de Clínicas of University of Campinas (HC-UNICAMP). Each patient underwent two treatment phases: single-use dialyzers HD sessions, followed by 12 consecutive HD sessions with reuse of dialyzers. Data were collected at the following time points: single-use dialyzers sessions (N = 10 HD sessions), "1^st^," "6^th^," and "12^th^" reused dialyzers sessions (N = 30 HD sessions). A total of 40 HD sessions were analyzed in the study (Figure 1).

The inclusion criteria were age ≥ 18 years, diagnosis of CKD in HD treatment for at least 3 months, and sessions performed through arteriovenous fistula. Patients were selected by convenience and were reusing dialyzers prior to being switched to the first phase of the study (single-use dialyzers HD session). Demographic data collected were: age, ethnicity, gender, etiology and duration of renal disease, time on HD, and comorbidities. Patients with central venous catheter for HD, hepatitis B and C or HIV patients, who were immunosuppressed, clinically unstable, or presenting symptoms or signs suggesting infection or diagnosis of active infection were excluded. Patients who did not signed written informed consent due to disagreement about the proposed characteristics of hemodialysis sessions according to study definitions or due to personal reasons were not included.

Written informed consent was obtained from all patients and the local ethics committee approved the study protocol (CAAE 50735315.0.0000.5404); all clinical and research activities reported were consistent with the Declaration of Helsinki.

### Characteristics of hemodialysis sessions

Participants underwent 4-h HD sessions three times a week, through an arteriovenous fistula and a dialysate solution flow rate of 500 mL/min, with a standardized temperature according to the auricular (tympanic) temperature of the patient immediately before the HD session. Ultra filtration rate was set up until 10 mL/kg/h in each HD session. All the sessions were made with polysulfone dialyzer, at high flux and high efficiency, area of 1.8 m^2^, steam-sterilized by the manufacturer (HF 80S, Fresenius^(r)^). Considering the physical characteristics of the sample, there was no need for individualization of dialyzer surface area. The dialyzer brand was chosen based on the availability of the Nephrology Service. To avoid any impact on the microbiology of the dialysis fluid, the water for HD was treated by reverse osmosis and its microbiological quality was measured once a month (from September 2016 to February 2017).

### Clinical data collection, biochemical analysis, and dialyzers parameters

During the "single use" and "reuse" HD sessions, the following clinical parameters were collected at the beginning, hourly, and at the end: tremor, cyanosis, headache, urticarial reaction, axillary and auricular temperature, blood pressure, and heart rate. Blood samples for biochemical and microbiological analysis were collected at the beginning and/or at the end of the HD sessions according to Table 1. For endotoxinemia analysis, the Pierce LAL (Limulus Amebocyte Lysate) Chromogenic Endotoxin Quantitation Kit (N° 88282, Pierce, Rockford, IL, USA) was used, according to the manufacturer's instructions. The plate and dilution water were endotoxin-free. The correlation between absorbance and endotoxin concentration was linear in the 0.1-1.0 EU/mL range.

**Table 1:** Laboratory parameters collected pre- and post-hemodialysis sessions

Pre-hemodialysis	Post-hemodialysis
Urea	Urea
-	Urea reduction rate
-	kt/V
Albumin	Albumin
Hemoglobin	Hemoglobin
Hematocrit	Hematocrit
Ferritin	Ferritin
C-reactive protein	C-reactive protein
Endotoxinemia	Endotoxinemia
-	Blood culture
CLID/Endotoxins*	-
-	Residues of proteins in the dialyzer **
-	Residues of hemoglobin in the dialyzer **

* CLID: Culture and endotoxins screening in the dialyzer inner chamber fluid (blood chamber), after 48 to 72 h of reuse, just before the start of the hemodialysis session.

** After mechanical cleaning of the dialyzer with water treated by reverse osmosis under 20-25 psi pressure.

The process of dialyzers reuse was completely manual and performed by a trained professional. The procedure is set out in the Manual of Work Techniques of the Nephrology Service, which is based on the procedure for the reuse of dialyzers recommended by the Association for the Advancement of Medical Instrumentation (AAMI).^15^ After mechanical cleaning with water treated by reverse osmosis under 20-25 psi pressure, each dialyzer was filled with a sanitizing agent, according to manufacturer's instructions [0.2% peracetic acid, hydrogen peroxide, acetic acid, and active oxygen (Peroxide P50, Bell Type Industries, Brazil); dilution of the sanitizing agent was prepared using water treated by reverse osmosis. The dialyzers were stored in individualized and clean boxes, at room temperature, until next use (i.e., from 48 to 72 h later).

The information collected regarding the dialyzer included measurement of the priming volume and detection of protein and blood residues after mechanical cleaning; sample of liquid from the dialyzer blood chamber; and images of the dialyzer by naked eye inspection and by digital microscope.

Hemocheck-S (Pereg GmbH, Waldkraigburg, Germany) was used for the detection of blood residue, whose limit of detection is 0.1 µg of hemoglobin. Protein residue detection was performed using the Pyrrol-E (Pereg GmbH, Waldkraigburg, Germany) biopsy/endoscopes, instrumental and surface protein residues test, whose limit of detection is 1 µg of protein. Protein and blood residues in the dialyzer was assessed after mechanical cleaning for removal of blood and debris. The dialyzer model used in the study allows the opening of the blood compartment lid to access the fiber bundle at the entry and exit points for blood.

Liquid samples from the dialyzer inner chamber collected from 48 to 72 h after reuse and immediately before removal of the sanitizing agent at 1^st^, 6^th^ and 12^th^ (N = 30) reuse were subjected to the following analyses: concentration of heterotrophic bacteria and total coliforms (lower limit of quantification of 1.0 CFU/mL), and endotoxins quantification by the Limulus Amebocyte Lysate (LAL) PyrogentTM Plus (lower limit of quantification of 0.125 EU/mL).

The dialyzers images were taken using the USB 2.0 digital microscope (Plugable, Model: USB2-250X, China), at 250x magnification. Photographs of the blood inlet and outlet areas of the fibers were taken.

### Statistical analysis

Results were reported as means and SD or medians and interquartile ranges for continuous variables, or in frequencies and percentages for categorical variables. Normality of the variables was assessed using the Kolmogorov-Smirnov test. Average comparisons of continuous variables were made with the Student-t or Mann-Whitney tests. Comparisons of the variation (delta) between groups were made with the Wilcoxon, Kruskall-Wallis or ANOVA tests. Two-tailed P values < 0.05 were considered statistically significant. All statistical analyzes were performed using the SPSS program (version 17.0, SPSS Inc., Chicago).

## Results

The mean age of the 10 patients was 37 ± 16 years, 6 (60%) were male, and 5 (50%) were white. CKD and hemodialysis time was 169 ± 108 and 47 (23 - 111) months, respectively. Only 2 (20%) were diagnosed with diabetes mellitus, while 6 (60%) were hypertensive. Chronic glomerulonephritis was the cause of CKD in 8 (80%) patients. During hemodialysis sessions, patients used a heparin dose of 107 ± 34 IU/kg/session. The ultrafiltration rate was 2 ± 0.6, 2.2 ± 0.5, 2.0 ± 0.6 and 1.6 ± 1.0 liters for the single-use, 1^st^, 6^th^ and 12^th^ reuses, respectively.

Clinical and laboratory parameters for single-use sessions are detailed in Table 2 and for reuse sessions (1^st^, 6^th^ and 12^th^) are shown in Table 3. Serum C-reactive protein levels (CRP) [4.9 (2-14) mg/mL], ferritin (454 ± 223 ng/mL) and endotoxin [0.76 (0.61-0.91) EU/mL] were elevated at baseline and thus remained until the 12^th^ session. Although serum endotoxin levels were elevated, they did not increase significantly at the end of the HD session in both phases (i. e., single use or reuse).

**Table 2:** Clinical and laboratory parameters pre- and post-hemodialysis from patients who underwent sessions with "single-use" dialyzer

Parameters	Pre-HD (N = 10)	Post-HD (N = 10)	*p*
*Clinical manifestations*			
Heart rate (bpm)	68 ± 12	76 ± 18	0.07
MAP (mmHg)	95 ± 14	100 ± 21	0.41
Axillary temperature (ºC)	35.7 ± 0.3	35.4 ± 0.5	0.28
Auricular temperature (ºC)	36.2 ± 0.6	36.0 ± 0.4	0.30
*Biochemistry and hemodialysis*			
Albumin (g/dL)	3.8	4.4	0.005
Hemoglobin (g/dL)	11.6 ± 0.8	13.0 ± 1.3	0.005
Hematocrit (%)	35 ± 2.5	39 ± 4	0.005
Urea (mg/dL)	126 ± 31	33 ± 11	0.005
URR (%)	-	74 ± 5	NA
Kt/V	-	1.6 ± 0.3	NA
*Priming volume (mL)*	-	111 ± 4	NA
*Inflammation and endotoxinemia*			
C-reactive protein (mg/mL)	4.9 (2 - 14)	5.4 (2 - 17)	0.009
Ferritin (ng/mL)	454 ± 223	560 ± 277	0.005
Endotoxins (EU/mL)	0.76 (0.61-0.91)	0.83 (0.59-1.26)	0.12

MAP: mean arterial blood pressure; URR: urea reduction rate; NA: non-applicable.

**Table 3:** Clinical and laboratory parameters pre- and post-hemodialysis from patients who underwent sessions with reuse of dialyzers (1^st^, 6^th^ e 12^th^ sessions)

	1^st^ reuse			6^th^ reuse			12^th^ reuse		
	Pre-HD	Post-HD		Pre-HD	Post-HD		Pre-HD	Post-HD	
Parameters	(N = 10)	(N = 10)	*p*	(N = 10)	(N = 10)	*p*	(N = 10)	(N = 10)	*p*
*Clinical manifestations*									
Heart rate (bpm)	73 ± 12	72 ± 17	0.91	79 ± 16	75 ± 12	0.23	66 ± 10	76 ± 15	0.02
MAP (mmHg)	90 ± 19	100 ± 21	0.67	93 ± 13	89 ± 15	0.67	96 ± 11	91 ± 21	0.47
Axillary temperature (ºC)	35.9 ± 0.3	35.6 ± 0.6	0.15	35.9 ± 0.3	35.9 ± 0.3	0.503	36.0 ± 0.5	35.8 ± 0.5	0.12
Auricular temperature (ºC)	36.3 ± 0.3	36 ± 0.3	0.35	36.2 ± 0.2	36.3 ± 0.3	0.373	36.3 ± 0.4	36.0 ± 0.5	0.64
*Biochemistry and hemodialysis*									
Albumin (g/dL)	3.6 ± 0.4	4.3 ± 0.6	0.005	3.6 ± 0.5	4.3 ± 0.5	0.005	3.6 ± 0.4	4.2 ± 0.5	0.02
Hemoglobin (g/dL)	11.3 ± 0.8	12.7 ± 1.6	0.005	10.8 ± 1.3	12.3 ± 1.9	0.007	10.3 ± 1.4	11.5 ± 2.0	0.04
Hematocrit (%)	34 ± 2.8	38 ± 5	0.007	34 ± 3.8	36 ± 5.3	0.007	31 ± 4.2	34 ± 5.6	0.12
Urea (mg/dL)	112 ± 25	29 ± 13	0.005	123 ± 26	34 ± 16	0.005	114 ± 39	31 ± 16	0.008
URR (%)	-	75 ± 7	NA	-	73 ± 8	NA	-	72 ± 16	NA
Kt/V	-	1.7 ± 0.4	NA	-	1.6 ± 0.4	NA	-	1.6 ± 0.6	NA
* priming volume (mL)*	-	107.9 ± 3.9	NA	-	107.8 ± 4.1	NA	-	105.3 ± 6	NA
*Inflammation and endotoxins*									
C-reactive protein (mg/mL)	4.6 (1.6-16.7)	5.5 (2.2-17.9)	0.009	5.8 (1.9-15.5)	6.0 (2.3-22.6)	0.01	5.0 (1.7-13.6)	5.8 (2.1-13.1)	0.23
Ferritin (ng/mL)	440 ± 227	539 ± 286	0.005	617 ± 367	794 ± 363	0.07	514 ± 305	618 ± 357	0.02
Endotoxinemia (EU/mL)	0.82 (0.74-1.0)	0.94 (0.79-1.3)	0.11	-	-	-	0.67 (0.61-0.79)	0.67 (0.63-0.84)	0.75

MAP: mean arterial blood pressure; URR: urea reduction rate; NA: non applicable.

There was no significant clinical changes pre- and post-HD in the two phases of the study, except for the heart rate parameter at the 12^th^ reuse (66 ± 10 *vs*. 76 ± 15, *p* = 0.02).

**Figure 2:**
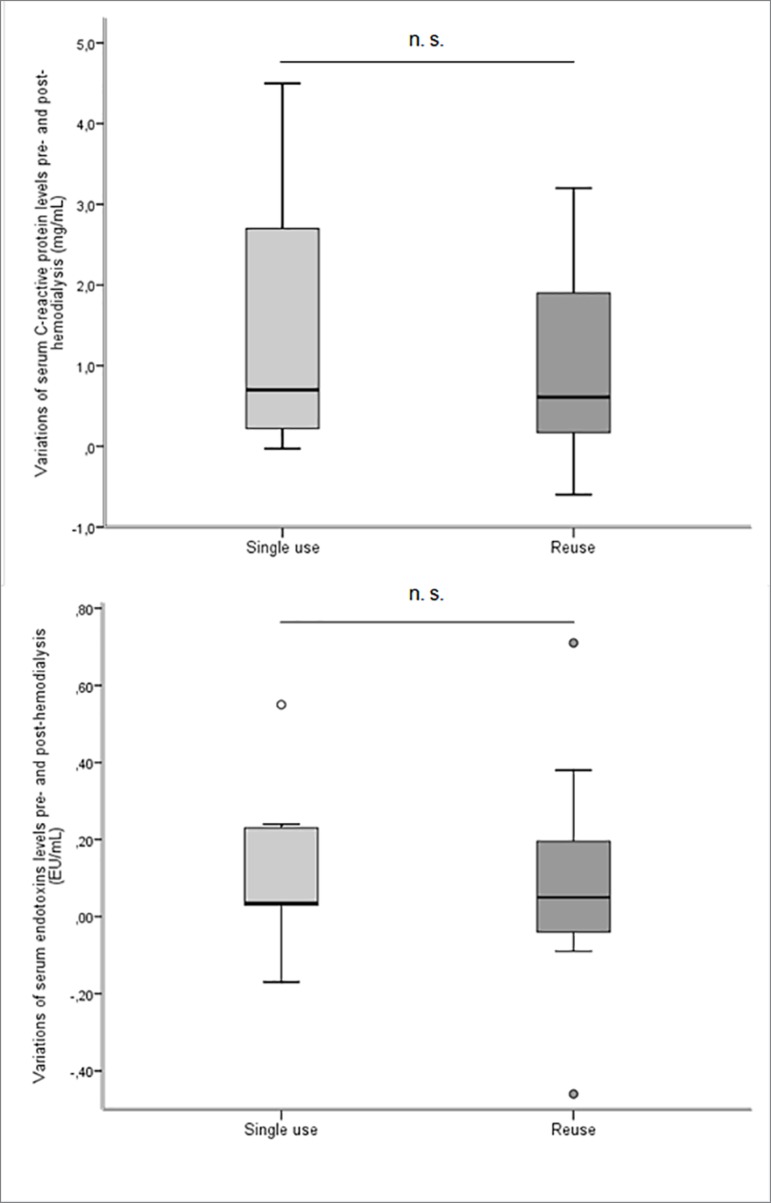
Comparisions of pre- and post-hemodialysis variations (delta) of serum C-reactive protein and endotoxins levels, between the "single use" and 'reuse" phases.

Comparison of pre- and post-hemodialysis variations (delta) between the "single use" and "reuse" phases. did not result in any significant difference in the parameters evaluated, including serum CRP (*p* = 0.8) and endotoxins levels (*p* = 0.4) (Figure 2). Likewise, the multiple comparison of these parameters, considering the "single use" and "1^st^, 6^th^ and 12^th^ reuse" phases did not result in significant differences (*p* = 0.6, *p* = 0.4, respectively).

Monthly bacterial culture and endotoxins analyses in HD water treated by reverse osmosis provided results within the recommended safety limits throughout the study (i.e., endotoxins levels < 0.25 EU/mL and heterotrophic bacteria count < 100 CFU/mL).

All samples collected from the sanitizing liquid stored in the dialyzer blood chamber were negative for the growth of total heterotrophic or coliform bacteria, as no endotoxins were detected.

Of the blood samples collected at the end of the HD sessions, 2 (20%) were positive in patients submitted to the single use sessions, whereas 1 was positive in the reuse phase, (at 1^st^ reuse). There was no relationship between the positivity of the test, clinical history, and physical examination, thereby excluding the possibility of non-dialysis related infections. These results were then interpreted as sample contaminations.

**Figure 3:**
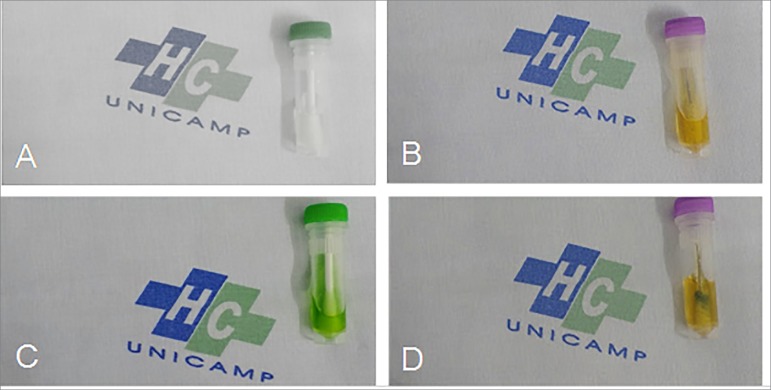
Detection tests for blood residues (Hemocheck-S) and proteins (Pyrmol-E) collected from reused dialyzers, after mechanical cleaning. Images A and B (negative controls) represent absence of blood and protein residues, respectively. Images C and D represent the presence of blood and protein residues, respectively.

**Figure 4:**
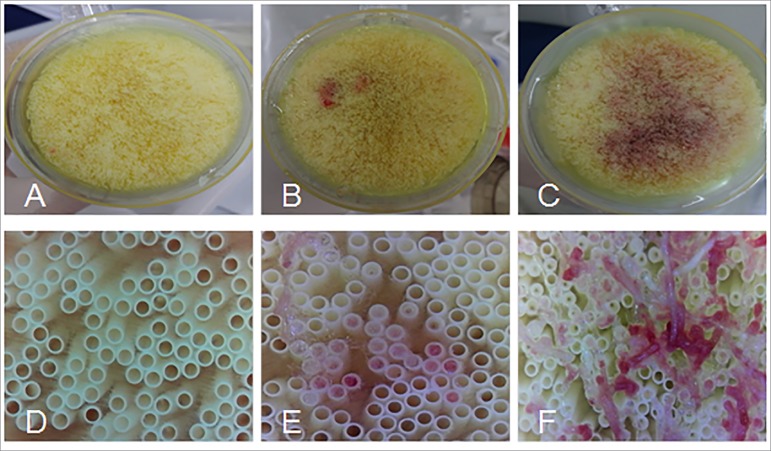
Images of dialyzer fibers after mechanical cleaning at different time points. Images A, B, and C, inspection with the naked eye; images D, E, and F, at 250x magnification. A and D, patient number 3 (1^st^ reuse); B and E, patient number 5 (6^th^ reuse); C and F, patient number 10 (12^th^ reuse).

Blood and protein residues were found in 5 to 9 (50 to 90%) and 6 to 10 (60 to 100%) of the 1^st^ and 12^th^ reuse sessions, respectively (Figures 3 and 4).

## Discussion

Our study had three main results: first, the reuse of dialyzers was safe from the microbiological point of view; second, no difference in clinical parameters or serum markers related to inflammation was observed between single-use with reuse phases; third, the dialyzer performance remained adequate until the 12^th^ reuse, considering the classic parameters of HD adequacy.

Scientific evidence points to negative effects of reusing dialyzers in terms of biological safety risk and mortality,^4^^,^^8^^-^11 although there is conflicting evidence.^16^ It should be noted that most studies have low quality and are performed with non-biocompatible dialyzers that are based on cellulose or modified cellulose.^7^^,^^17^^-^19 Also, the control of reprocessing dialyzer methods is difficult to carry out over time, especially if strict protocols are not followed.

Although we did not observe an impact on serum markers of inflammation with the reuse phase compared to single use (ie, serum levels of CRP and ferritin), some authors believe that patient exposure to denatured blood products from degradation of protein and hemoglobin may result in increased oxidative stress.^20^ Their long-term impacts are not known.

In our study, we did not detect significant differences between serum levels of pre- and post-HD endotoxins between the two phases, although a lower level of post-HD endotoxins at the reuse phase compared to the single-use phase was detected [0.67 (0.63-0.84) *vs*. 0.83 (0.59-1.26) ]. Vanholder et al. observed that large pore dialyzers favor the transfer of endotoxins through the back diffusion/filtration phenomenon.^21^ The potential reduction of medium molecules clearance attributed to the reuse process^22^ may play a role in the attenuation of endotoxin transfer.

In theory, the single-use dialyzers are related to a lower probability of bloodstream infection and reduction of medium molecules clearance, in addition to decrease risk of exposure to denatured blood products. In addition, the sanitizing agents per se be might be a deleterious factor of dialyzer reuse. Port et al. followed 12.791 hemodialysis patients for 1 to 2 years and concluded that although mortality was not higher in patients exposed to reuse of dialyzers than to single-use ones, there may be differences in mortality risk attributed to the type of sanitizing agent used for the reuse of dialyzer.^23^ Solutions based on peracetic acid and formaldehyde may be more harmful to patients when compared to the use of chlorine-based substances or to heat as a forms of disinfection.^24^

However, as there is no robust evidence on outcomes, costs, and environmental impact, and given the increasing economic pressure on the health systems predicted for the coming decades, we believe that these issues still need to be answered prior to establishing the culture of single-use dialyzers. Some authors argue that in several countries the reuse of dialyzers allow more patients on hemodialysis.^5^^,^^6^

This pilot study had limitations. The number of HD sessions, patients, and the follow-up time are restricted. The sample was constituted mainly by young patients with chronic glomerulonephritis as CKD etiology due to the intrinsic characteristics of our institution, not properly representing the epidemiology of CKD causes. The use of inflammatory markers such as CRP and ferritin may not adequately demonstrate inflammatory activation, and we did not evaluate the impact of single-use or reusable dialyzers in other parameters, such as interleukins 6 and 18. Patients did not go through a washout phase before starting the first phase of the study, which may affect the serum inflammation parameters. Although data such as the kt/V and urea reduction rate remained adequate during the reuse phase, we did not assess the efficiency of the dialyzer in the removal of medium molecules.

In conclusion, our results indicated that reuse of dialyzers have similar characteristics to single-use ones in terms of biological risk, inflammation activation, and dialyzer efficiency assessed by classical urea kinetics parameters. Such findings should be confirmed in larger studies that include the analysis of cost and environmental impact associated with dialyzers reuse practices.
